# Intrinsic Differential Scanning Fluorimetry for Protein Stability Assessment in Microwell Plates

**DOI:** 10.1021/acs.molpharmaceut.4c01496

**Published:** 2025-02-07

**Authors:** Michaela Cohrs, Alastair Davy, Manon Van Ackere, Stefaan De Smedt, Kevin Braeckmans, Markus Epe, Hristo L. Svilenov

**Affiliations:** † Laboratory of General Biochemistry and Physical Pharmacy, Ghent University, Ottergemsesteenweg 460, Ghent 9000, Belgium; ‡ Protein Stable Ltd.,, 21 Mole Business Park, Leatherhead KT22 7BA, U.K.; § Biopharmaceutical Technology, TUM School of Life Sciences, Technical University of Munich, Emil-Erlenmeyer-Forum 5, Freising 85354, Germany

**Keywords:** differential scanning fluorimetry, modulated scanning fluorimetry, biotherapeutics, monoclonal antibodies, intrinsic fluorescence, thermal stability

## Abstract

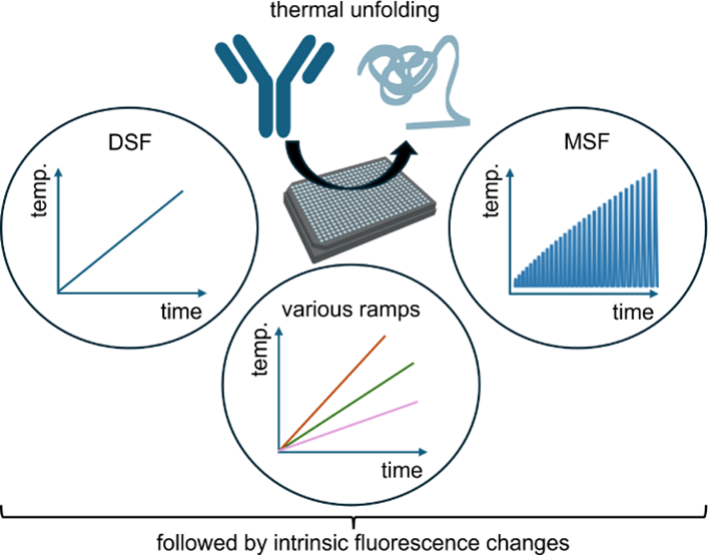

Intrinsic differential scanning fluorimetry (DSF) is essential for analyzing protein thermal stability. Until now, intrinsic DSF was characterized by medium throughput and high consumable costs. Here, we present a microplate-based intrinsic DSF approach that enables the measurement of up to 384 samples in parallel by consuming only 10 μL per sample. We systematically test and benchmark the new intrinsic DSF against gold-standard methods such as differential scanning microcalorimetry and circular dichroism. Using a range of model proteins and sample conditions, we demonstrate the robustness and versatility of the intrinsic DSF method for characterizing protein stability and ranking protein drug candidates. In addition, we demonstrate modulated scanning fluorimetry (MSF) capabilities on the intrinsic DSF hardware that enable simultaneous MSF measurements in 384-microwell plates. Overall, the presented technology is a powerful tool for the early stability analysis of various protein samples and drug candidates.

## Introduction

High thermostability is a desired attribute of protein drug candidates.^1,2^ Differential scanning fluorimetry (DSF) has become invaluable for analyzing protein thermal stability very early during drug development.^3,4^ In general, DSF experiments are either performed with a reporter dye (i.e., extrinsic DSF) or dye-free (i.e., intrinsic DSF).^2,5,6^ In the intrinsic approach, the fluorescence of aromatic amino acids (mainly Trp) is measured as a function of temperature. Because the spectral properties of Trp are sensitive to the microenvironment, structural changes (e.g., unfolding) in Trp-containing proteins can be detected.^7^

Different types of thermostability experiments can be performed by varying the heating program or protein concentration. The classical DSF experiments employ fixed linear heating rates (e.g., 1 °C/min) that yield well-known parameters such as protein unfolding onset (T_onset_) and (apparent) melting temperatures (*T*_m_s).^2,8,9^ In addition, DSF can be used to determine a number of orthogonal stability-indicating parameters such as the activation energy (*E*_a_)^10^ or the Δ*T*_m_ shift.^11,12^ Moreover, isothermal DSF at elevated temperatures or experiments with different heating rates can provide additional insights into the kinetics of protein unfolding and aggregation.^13,14^ Last but not least, intrinsic DSF can be applied to detect stabilizing protein interactions and estimate binding affinities of protein ligands.^15−18^

In addition, there are new techniques using intrinsic DSF hardware. For example, modulated scanning fluorimetry (MSF) applies incremental heating and cooling cycles to probe the reversibility of structural changes induced by a short exposure to different temperatures.^16,17^ With MSF, the temperature that starts to cause irreversible structural changes (T_nr_) can be determined.^16,17^

Despite the increasing importance of intrinsic DSF, this technique has been almost exclusively performed in glass capillaries or (micro)cuvettes. As a result, intrinsic DSF is associated with limited throughput, high consumable costs and automation challenges.

Here, we present an intrinsic DSF methodology for protein stability assessment in microwell plates. We demonstrate that the platform is capable of robust label-free analysis of various protein drug modalities such as antibodies, fusion proteins, hormones and enzymes. We benchmark the intrinsic DSF against gold standard methods and demonstrate the compatibility of the technique with various sample conditions to study protein stability in the presence of surfactants, impurities and ligands. In addition, we demonstrate the possibility of MSF measurements of up to 384 samples in parallel.

## Materials and Methods

### Model Proteins

The following proteins were used in this study: eight IgG1κ from commercial drug products and eight (pre)clinical-stage molecules (three IgG1κ, one IgG1λ, one IgG2κ, three IgGs with engineered Fc regions). In addition, we included one albumin-fusion protein, one ACE2-IgG-Fc fusion protein, recombinant human growth hormone (rHGH) and hen-egg white lysozyme (HEWL). The excipients from the commercial products were removed by cation exchange chromatography. Dialysis was used to exchange the formulation buffers. All chemicals used were pharmaceutical grade or higher.

### Intrinsic Differential Scanning Fluorimetry

Unless otherwise stated, black 384-well, polypropylene, PCR plates (HSP3866, Bio-Rad, Hercules, USA) were filled with 10 μL sample and sealed with qPCR adhesive seals (4ti-0560, Azenta, Burlington, USA) before centrifugation for 2 min at 2000 rpm in a benchtop centrifuge (Heraeus multifuge 1 S-R, Germany). As a comparison, white polypropylene plates were used (HSP3805, Bio-Rad, Hercules, USA) and the filling volumes were varied. The measurements were performed with a SUPR-DSF device (Protein Stable, Leatherhead, UK) using a linear heating ramp. The samples were excited at 280 nm and the emission spectra were collected from 310 to 420 nm. To obtain the thermal unfolding curves, the barycentric mean (BCM) of the emission spectra was calculated and plotted against the temperature. The melting temperatures were fitted from the unfolding curve using the SUPR-Suite software (version 1.1.2.1).
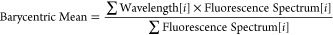


### Isothermal Fluorimetry

The same hardware was used for isothermal fluorescence data. A Python script was used to obtain the BCM of antibody samples over the course of 24 h at 50, 55, and 60 °C. Scans were taken every 15 min.

### Modulated Scanning Fluorimetry (MSF)

The MSF measurements were conducted using the same hardware, with an AutoIT script to automate the control of the hardware, while a custom-made Python script was used to collect and process the measurement files. We conducted cycles consisting of 5 min holding at 25 °C before heating (10 °C/min) to the target temperature followed by 1 min hold time and cooling to 25 °C. The target temperature was increased by 1 °C for each cycle up to 105 °C. Data analysis was performed by normalization of unfolding and nonreversibility curves using the BCM in Origin Pro 2024 (Northampton, USA). T_nr_ was defined as the 10% offset from the baseline of the nonreversibility curve. For measurements with a baseline noise or shift >10%, the baseline was determined manually.

### Differential Scanning Microcalorimetry (μDSC)

Three model proteins were characterized on a MicroCal PEAQ-DSC system (Malvern Panalytical, Malvern, UK). All samples were measured as duplicates of 250 μL (1 mg/mL) against their respective buffer. A heat ramp of 1 °C/min was applied in the range from 20 to 100 °C. The thermograms of the buffer were subtracted for peak analysis. The melting temperatures were derived from the peak maxima.

### Far UV Circular Dichroism (FUV CD)

Thermal transitions of the three model proteins were measured via FUV CD on a Chirascan CD spectrometer (Applied Photophysics, Leatherhead, UK) in triplicates. A thermal ramp of 1 °C/min was applied from 20 to 90 °C and the CD signal was measured at 205 nm for ADAL and BEVA and 222 nm for HEWL. Protein concentration was 0.1 mg/mL. The sample volume of 300 μL was placed in a 1 mm quartz cuvette (Hellma Analytics, Müllheim, Germany). Unfolding curves were fitted with Boltzmann fit to determine the minimum or maximum of the first derivative.

### Extrinsic DSF with SyproOrange

Extrinsic DSF was performed on CFX384 Touch Real-Time PCR Detection System with C1000 Touch Thermal Cycler (Bio-Rad, Hercules, USA). Nineteen μL of model protein (1 mg/mL) were mixed with 1 μL of 50x Sypro Orange in DMSO. Three replicates each were pipetted in black 384-Well PCR plates (HSP3866, Bio-Rad, Hercules, USA) and sealed with a PCR plate sealing film (MSB1001, Bio-Rad, Hercules, USA). After centrifugation, a heat ramp of 1 °C/min was applied from 20 to 90 °C. The fluorescence was acquired every 1 °C using the FRET channel. Minima of the first derivative were used for *T*_m_ determination.

### Intrinsic Protein Fluorescence in Quartz Cuvettes

The intrinsic protein fluorescence was measured on a Jasco FP-8550 using a 10 × 2 mm quartz cuvette (Hellma Analytics, Müllheim, Germany). The spectra (300–450 nm) were collected at different temperatures after excitation at 280 nm. The measurements were performed in triplicates.

## Results

### Versatility and Robustness of Intrinsic DSF in Microwell Plates

**Figure 1:**
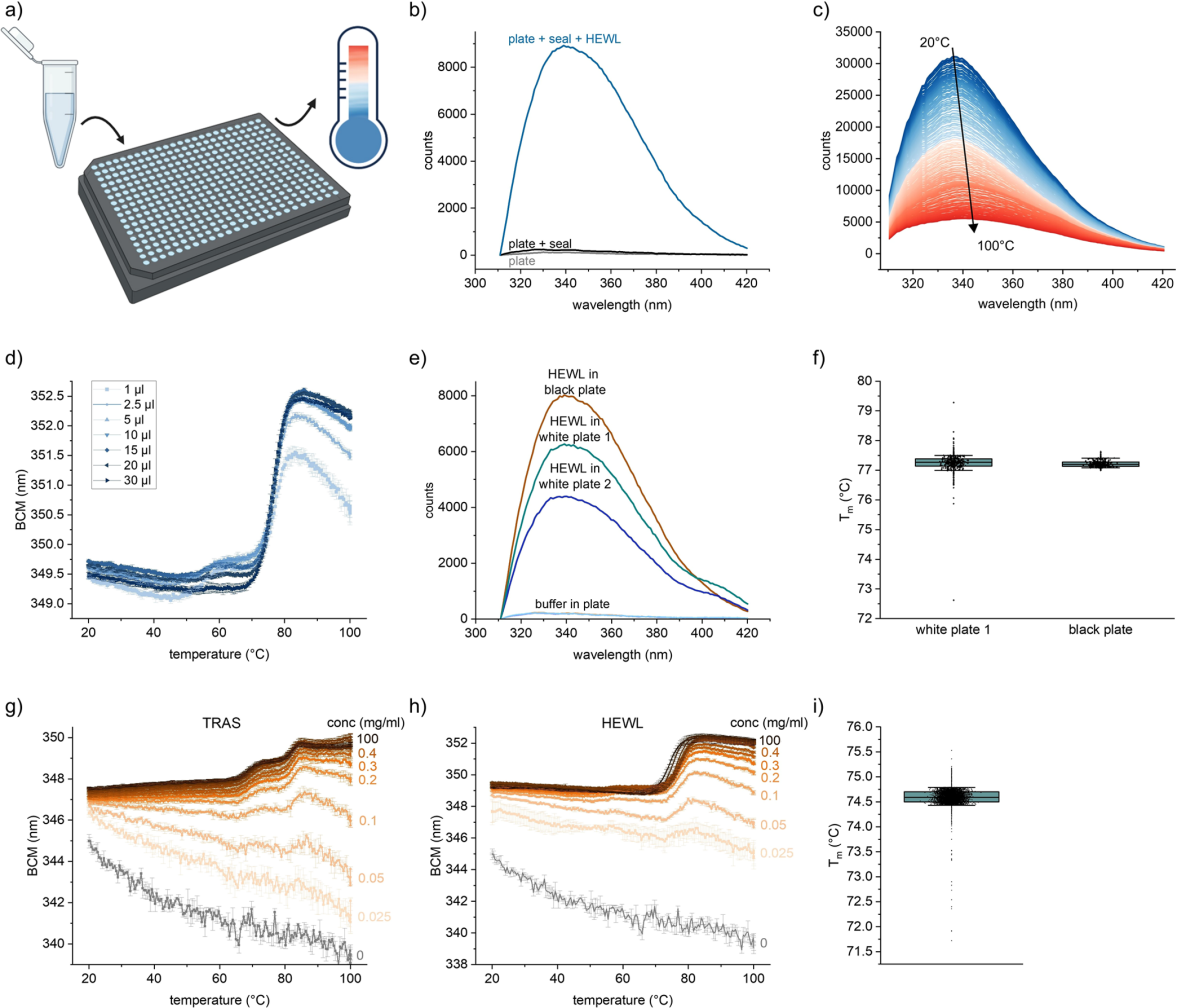
Versatility and robustness of intrinsic DSF in microwell plates. (a) Schematic experimental workflow. (b) Fluorescence emission spectra of the plate, plate with seal and plate with seal filled with 10 μL of 1 mg/mL HEWL (20 mM acetate buffer, pH 5) at 20 °C. (c) Exemplary spectral change collected during heating of a monoclonal antibody, TRAS. (d) Unfolding curves of HEWL 1 mg/mL in 20 mM acetate buffer, pH 5 determined with different sample volumes. Mean of triplicates with SD. (e) Fluorescence emission spectra of HEWL 1 mg/mL in 20 mM acetate buffer, pH 5 and buffer filled wells (background) for black and white polypropylene plates at 20 °C. (f) *T*_m_ values of HEWL determined in a full 384 well-plate. Left: white plate 1 (Bio-Rad). Right: black plate (Bio-Rad). All values are displayed. The box presents 25th and 75th percentile. The whiskers present 10th and 90th percentile. Outliers are shown in a row and were defined with Tukey’s Fences method with outliers below Q1 – 1.5 × interquartile range or above Q3 + 1.5 × interquartile range. (Q1 and Q3 are first and third quartiles). (g) Unfolding curves obtained with different concentrations of a model monoclonal antibody, TRAS. (h) Unfolding curves obtained with HEWL. Both measured in 20 mM acetate buffer pH 5. Mean of triplicates with SD. (i) Repeatability of *T*_m_ determined for 6096 measurements of HEWL at 0.5 mg/mL, pH 5.7. All values are presented. The box represents the 25th and 75th percentile. The whiskers show the 10th and 90th percentile. Outliers are presented in a row and were determined by Tukey’s Fences method.

The intrinsic DSF is performed in standard 384-microwell PCR plates (Figure 1a). The intrinsic protein fluorescence spectra from each well can be obtained, and the background signals from the plate and the plate seal are negligible (Figure 1b). The intrinsic fluorescence spectra obtained in PCR plates are comparable to the spectra obtained in quartz cuvettes (Figure S1a–e). Small differences between the spectra could be attributed to the different hardware optics and parameter variations between the instruments. The intrinsic DSF method allows the collection of intrinsic protein fluorescence spectra from every individual well during heating (Figure 1c). Our preliminary tests showed that data can be collected using different sample volumes (Figure 1d), although the instrument optics are optimized for a fill volume of 10 μL sample per well. Next, we wondered if the material of the PCR microwell plates has an influence on the data. Black polypropylene (PP) plates allow the measurement of higher intrinsic protein fluorescence intensity combined with less signal artifacts around 400 nm in contrast to white PP microwell plates (Figure 1e). In addition, the variation of the determined melting temperatures is smaller in black PP plates compared to white PP plates (Figure 1f).

A wide range of protein concentrations can be measured within the same run as illustrated with a range of HEWL and mAb concentrations from 0.025 to 100 mg/mL. Protein unfolding transitions can be detected already at around 0.05 to 0.1 mg/mL protein concentration (Figure 1g,h).

After defining these general features of the technique, we were interested in the variability of the method. To this end, we measured 6096 samples of HEWL (0.5 mg/mL, pH 5.7). The melting temperature was accurately determined as 74.6 °C with a standard deviation of 0.17 °C. The 10th and 90th percentile differ by less than 0.5 °C (Figure 1i). A total of 39 outliers (0.6%) were identified using the modified Z-score method (|Z|> 3.5).

Overall, these measurements reveal that the intrinsic DSF in microwell plates is a versatile and robust technique that allows the measurement of up to 384 protein samples with different concentrations in parallel.

### Comparing Intrinsic DSF in Microwell Plates with Gold-Standard Methods

**Figure 2:**
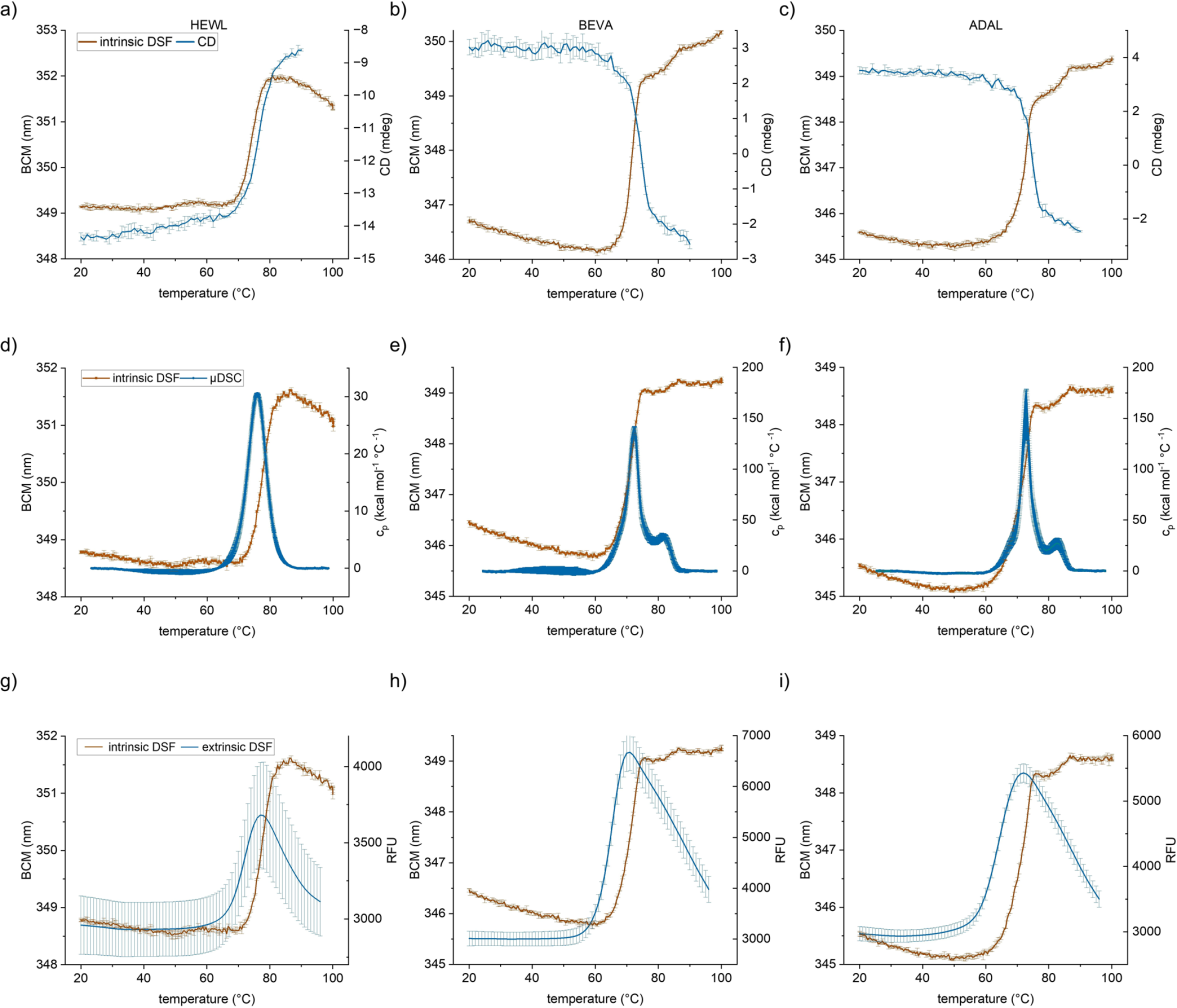
Comparability of intrinsic DSF in microwell plates with gold-standard methods. (a–c) Unfolding curves of HEWL, BEVA and ADAL in 10 mM phosphate buffer pH 6.5 determined with intrinsic DSF (1 mg/mL) and CD (0.1 mg/mL). Mean of triplicates with SD. (d–f) Unfolding curves of HEWL, BEVA and ADAL at 1 mg/mL in 20 mM acetate buffer pH 5 obtained with intrinsic DSF and μDSC. Mean of triplicates (DSF) and duplicates (μDSC) with SD. (g–i) Unfolding curves of HEWL, BEVA and ADAL at 1 mg/mL in 20 mM acetate buffer pH 5 obtained with intrinsic and extrinsic DSF. Mean of triplicates with SD.

We wondered how the intrinsic DSF in PCR plates compare to classical methods for protein stability analysis. Therefore, we analyzed the thermal unfolding of HEWL and two monoclonal antibodies using intrinsic DSF, differential scanning microcalorimetry (μDSC) and circular dichroism (CD). There is a very good agreement between the data obtained with the different techniques (Figure 2a–f and Table 1). For example, intrinsic DSF reveals two unfolding transitions in each of the antibodies that are also detected by μDSC. In comparison to μDSC, the intrinsic DSF has the major advantage of requiring only 10 μL of sample and being able to measure up to 384 samples in parallel.

**Table 1:** Apparent Melting Temperatures Determined by Intrinsic DSF, CD, μDSC and Extrinsic DSF^a^^b^^c^

	HEWL	BEVA		ADAL	
Method (sample buffer)	*T*_m_ (°C)	T_m1_ (°C)	T_m2_ (°C)	T_m1_ (°C)	T_m2_ (°C)
**Intrinsic DSF (phosphate)**	74.21 ± 0.04	71.57 ± 0.07	84.33 ± 1.07	72.47 ± 0.04	84.11 ± 0.05
**CD (phosphate)**	76.25 ± 0.09	73.50 ± 0.14	n.d.	74.22 ± 0.31	n.d.
**Intrinsic DSF (acetate)**	77.50 ±0.15	70.95 ± 0.10	83.59 ± 0.73	71.17 ± 0.13	84.02 ± 0.63
**μDSC (acetate)**	76.01 ± 0.22	72.07 ± 0.00	81.15 ± 0.03	72.72 ± 0.04	81.83 ±0.03
**Extrinsic DSF (acetate)**	72 ± 0	65.67 ±0.47	n.d.	64 ± 0	n.d.

a Mean of triplicates for intrinsic μDSF, CD and extrinsic DSF with SD.

b Mean of duplicates with SD for μDSC.

c Samples in 20 mM phosphate buffer pH 6.5 or 20 mM acetate buffer pH 5.

Another well-established method for protein thermal stability analysis is extrinsic DSF in PCR plates using fluorescent dyes such as SyproOrange. We tested how the intrinsic and extrinsic DSF approaches compare to each other. Interestingly, the unfolding curves from extrinsic DSF are shifted to lower temperatures compared to intrinsic DSF (Figure 2g–i). As a result, the apparent melting temperatures from the extrinsic DSF are lower compared to intrinsic DSF (Table 1). A possible explanation of these differences is that the fluorescence of the extrinsic dye changes considerably already in the presence of lower fractions of unfolded protein. In contrast, the change in the intrinsic fluorescence likely requires a larger structural change in the protein.

**Figure 3:**
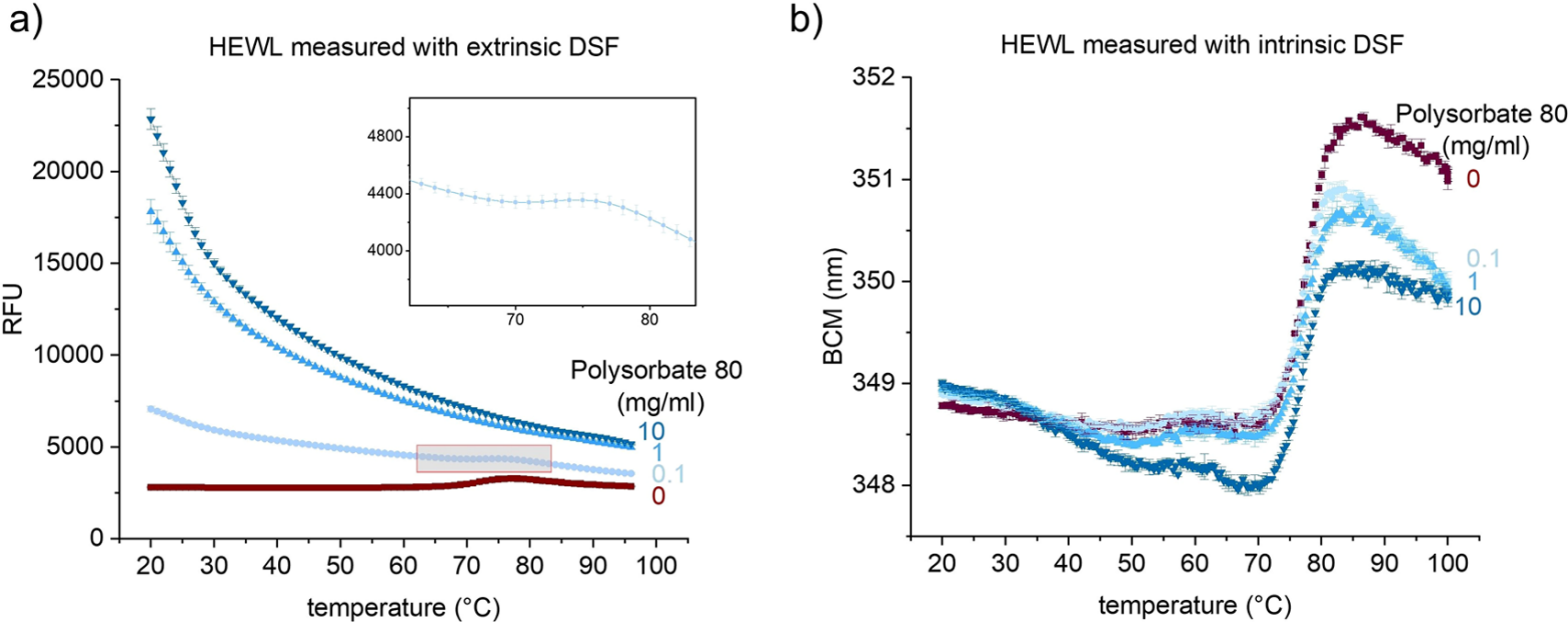
Suitability of extrinsic and intrinsic DSF in microwell plates for analysis of protein samples containing a surfactant. Fluorescence data obtained with HEWL 1 mg/mL in 20 mM acetate buffer pH 5 in the presence of 0 to 10 mg/mL polysorbate 80. (a) Extrinsic DSF with SyproOrange. (b) Intrinsic DSF. Mean of triplicates with SD.

In addition, the extrinsic DSF employing SyproOrange is not compatible with most routinely used surfactants. To test whether the intrinsic DSF performs better on samples containing a surfactant, we performed intrinsic and extrinsic DSF measurements of HEWL in the presence of varying polysorbate concentrations. Thermal unfolding curves can be obtained at all polysorbate concentrations with intrinsic DSF (Figure 3b) while already 0.1 mg/mL polysorbate increased the SyproOrange fluorescence and almost completely masked the protein unfolding transition (Figure 3a). Therefore, intrinsic DSF has a significant advantage over SyproOrange-based DSF when measuring samples with surfactants.

### Impact of Sample Impurities on Intrinsic DSF Data

During the early development stages, protein samples can contain a range of impurities such as nucleic acids, residual solvents or chelating agents. Therefore, we tested if intrinsic DSF can be used to assess protein stability in samples containing impurities. Specifically, we measured the stability of HEWL in different concentrations of EDTA, DMSO and pDNA.

**Figure 4:**
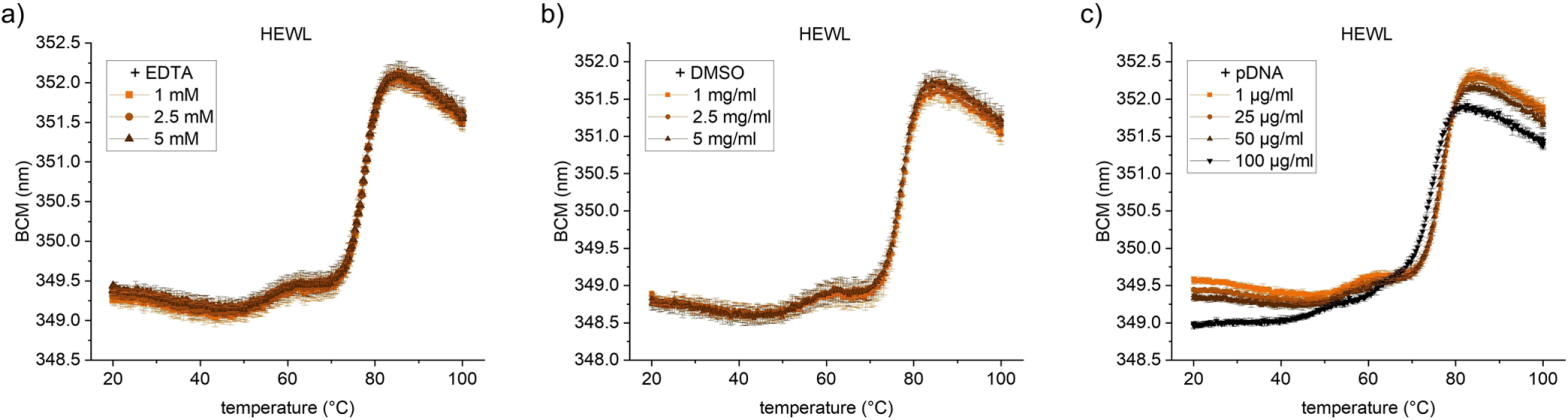
Robustness of DSF in microwell plate towards impurities. Unfolding of 1 mg/mL HEWL in 20 mM acetate buffer, pH 5 under addition of (a) EDTA, (b) DMSO and (c) pDNA. Mean of triplicates with SD.

None of these compounds changed the background (Figure S2) and neither impacted the unfolding curves of HEWL (Figure 4). Only at high DNA concentrations (100 μg/mL), there is a slight shift in the baseline of the curve and a slight reduction of the apparent melting temperature of HEWL (Figure 4c).

### Intrinsic DSF for Ranking of Antibody Drug Candidates

**Figure 5:**
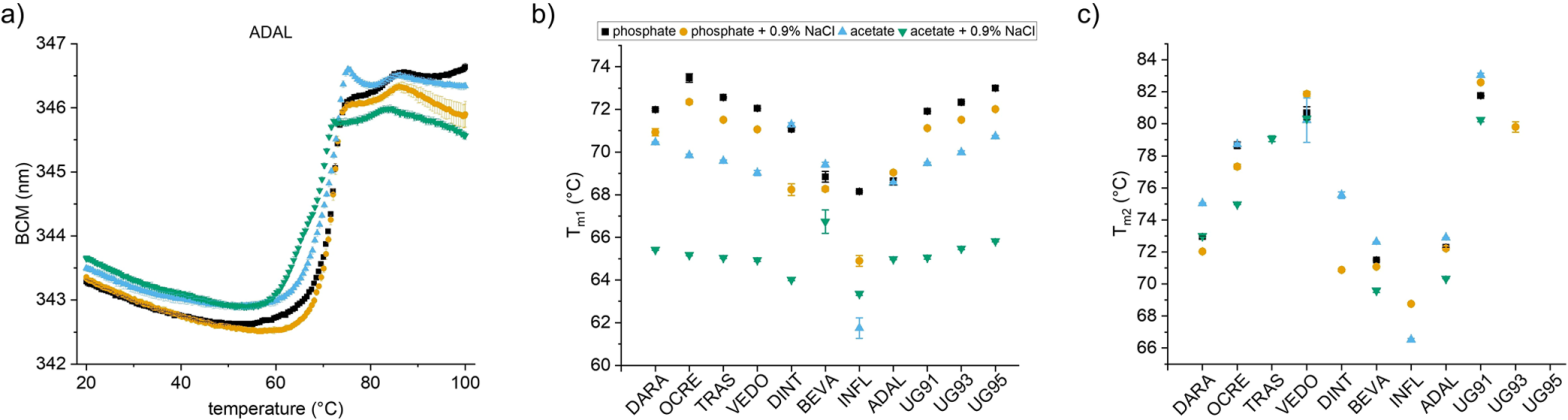
Antibody drug candidate profiling with intrinsic DSF in microwell plates. (a) Exemplary unfolding curves of a model antibody (ADAL) in four conditions. (b) T_m1_ and (c) T_m2_ determined for the IgG1k across the four conditions. Mean of triplicates with SD.

One of the major applications of DSF is in the developability assessment of antibody drug candidates. Therefore, we were interested in whether this technology can be applied to various antibodies and formulations. To this end, we formulated a set of IgG1κ antibodies in four relevant conditions (10 mM sodium acetate pH 5 and 10 mM phosphate pH 7 with or without 0.9% NaCl) and performed classical intrinsic DSF measurements with a fixed heating rate (1 °C/min). The thermal unfolding curves revealed substantial differences between the antibodies and different formulations (Figures 5a and S3). We could determine at least one melting temperature for all antibodies (Figure 5b). For most of the antibodies and conditions, we could also determine a second melting temperature (T_m2_) consistent with the expected behavior of multidomain proteins (Figure 5c).^19^ For some antibodies, three melting temperatures could be resolved that could be attributed to the Fab, C_H_2 and C_H_3 domains. Overall, the antibodies showed higher thermal stability in phosphate pH 7 compared to acetate pH 5 which is expected for IgG1κ.^20^ The lowest thermal stability was measured in acetate pH 5 with 0.9% NaCl.

### Intrinsic DSF for Characterization of Different Protein Modalities

**Figure 6:**
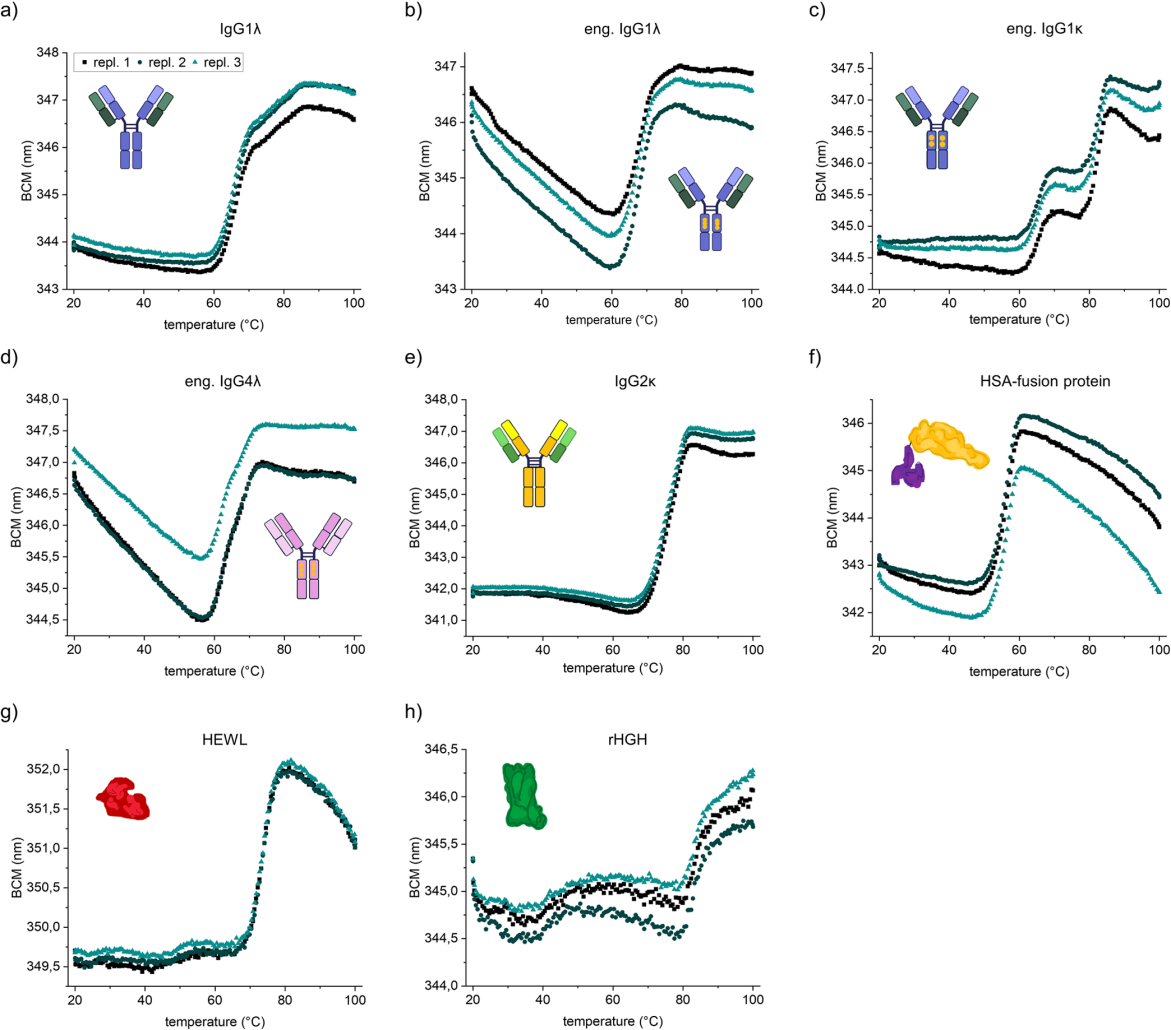
Different unfolding profiles of various protein modalities. All proteins are formulated at 0.5 mg/mL in 10 mM phosphate buffer, pH 7. Overlay of three replicates.

In addition to classical IgG1 antibodies, new protein modalities based on different antibody classes, engineered Fc regions, and fusion proteins become increasingly important. We therefore tested if the intrinsic DSF method can be applied to study various therapeutic protein modalities (Figure 6). Different unfolding profiles reveal differences in thermal stability. For multidomain proteins, multiple transitions can be resolved depending on the differences in the stability of the individual domains (Figure 6a–f). Single-domain proteins such as HEWL and hHGH yield only one unfolding transition (Figure 6g,h). Overall, the data shows that the microwell-plate-based intrinsic DSF can be applied to proteins with different sizes and complexities.

### Intrinsic DSF for Analysis of Ligand-Induced Protein Stabilization

**Figure 7:**
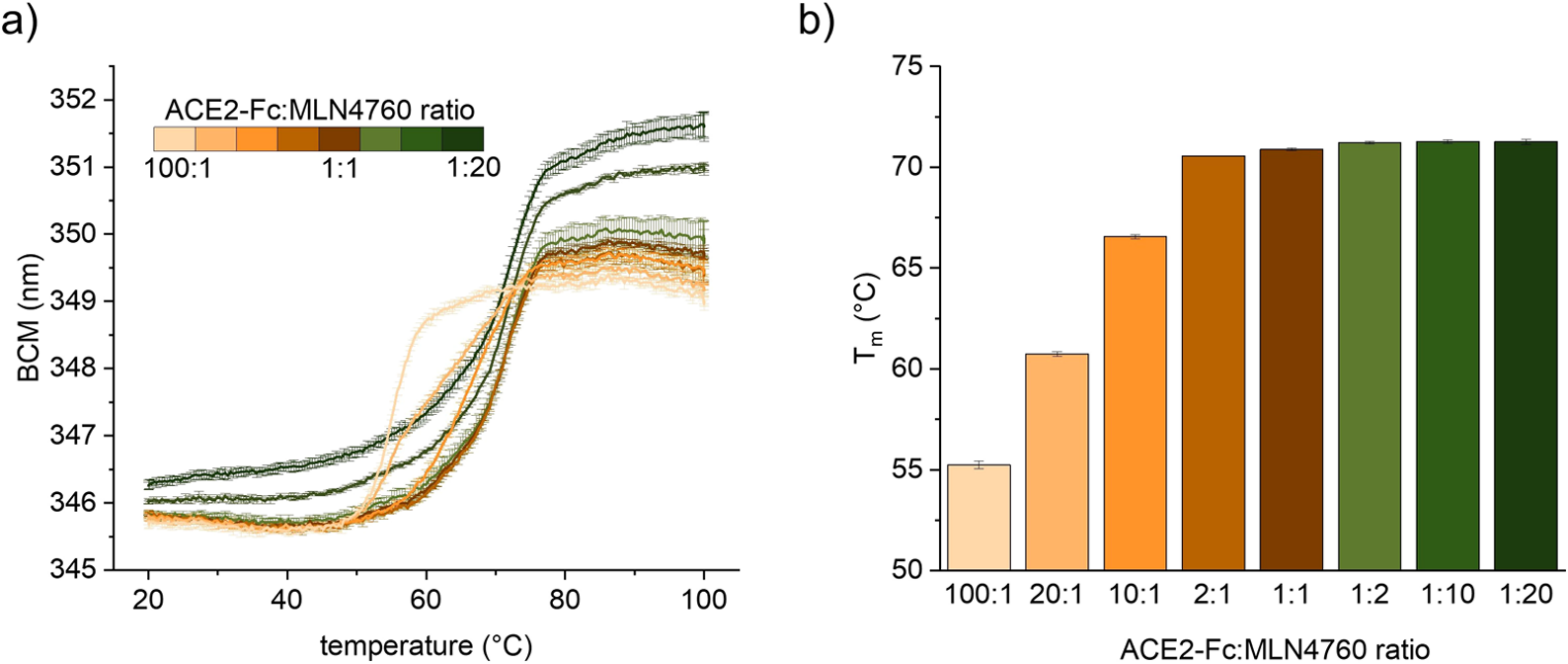
Stabilization of ACE2-Fc fusion protein by MLN4760. (a) Unfolding curves of ACE2-Fc fusion protein (0.5 mg/mL) in the presence of different molar ratios of MLN4760. Formulation in 1× PBS, pH 7.4. Mean of triplicates with SD. (b) Increase of T_m_ under the addition of MLN4760. Molar ratios of ACE2-Fc fusion protein: MLN4760 are shown. Mean of triplicates with SD.

In addition to studying the stability of protein drug candidates, thermal stability analysis can be used to screen for ligands (e.g., enzyme inhibitors) that increase the stability of a protein upon binding. To test whether intrinsic DSF can detect ligand-induced protein stabilization, we measured different molar ratios of an ACE2-Fc fusion protein and a small molecule ACE2-inhibitor (MLN4760) that increases the stability of ACE2 as observed by μDSC measurements.^21^ The apparent melting temperature of the ACE2 domain shifts to higher temperatures when the concentration of MLN4760 is increased. The maximum stabilization is reached at a molar ratio of 1:2 (ACE2-Fc:MLN4760) (Figure 7a,b).

### Heating Ramp and Protein Concentration Variation in Intrinsic DSF

Intrinsic DSF experiments performed at different heating rates could provide additional layers of information about the kinetics of thermal unfolding and aggregation. Therefore, we tested how the heating rate affects the unfolding onset temperatures of two model proteins – HEWL and rHGH.

**Figure 8:**
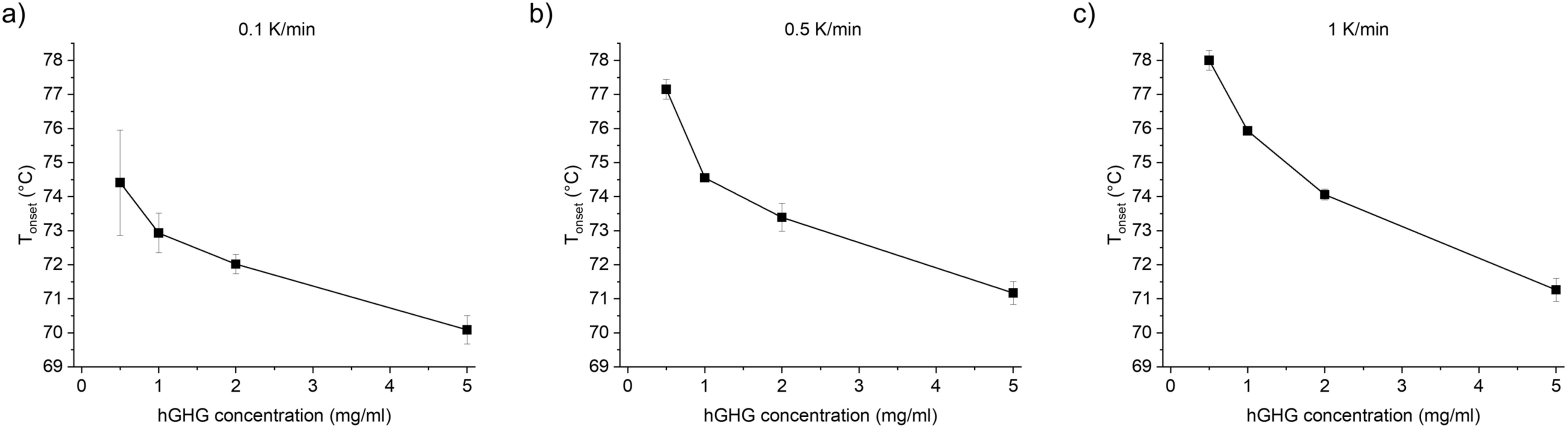
Differences in T_onset_ of rHGH caused by variations in ramp rate and protein concentration. 0.5–5 mg/mL rHGH in 10 mM phosphate buffer pH 7 measured with a ramp rate of (a) 0.1 K/min, (b) 0.5 K/min, and (c) 1 K/min. All values are mean of triplicates with SD.

The unfolding onset temperature of HEWL shows little dependence on the protein concentration and the heating rate (Figure S4a–c). The reason for this is that HEWL does not exhibit substantial aggregation during heating at these conditions that will affect the apparent melting temperatures.^16^ In contrast, the unfolding onset temperature of rHGH is very sensitive to the protein concentration and the heating rate, likely because rHGH is very prone to aggregation which has an impact on the apparent T_onset_ values (Figure 8a–c).

### Modulated Scanning Fluorimetry in Microwell Plates

MSF is based on the hardware for intrinsic DSF. MSF provides information on the reversibility of structural protein changes induced by incremental heating and cooling cycles. Agile and precise temperature control is necessary for MSF experiments.^16^

**Figure 9:**
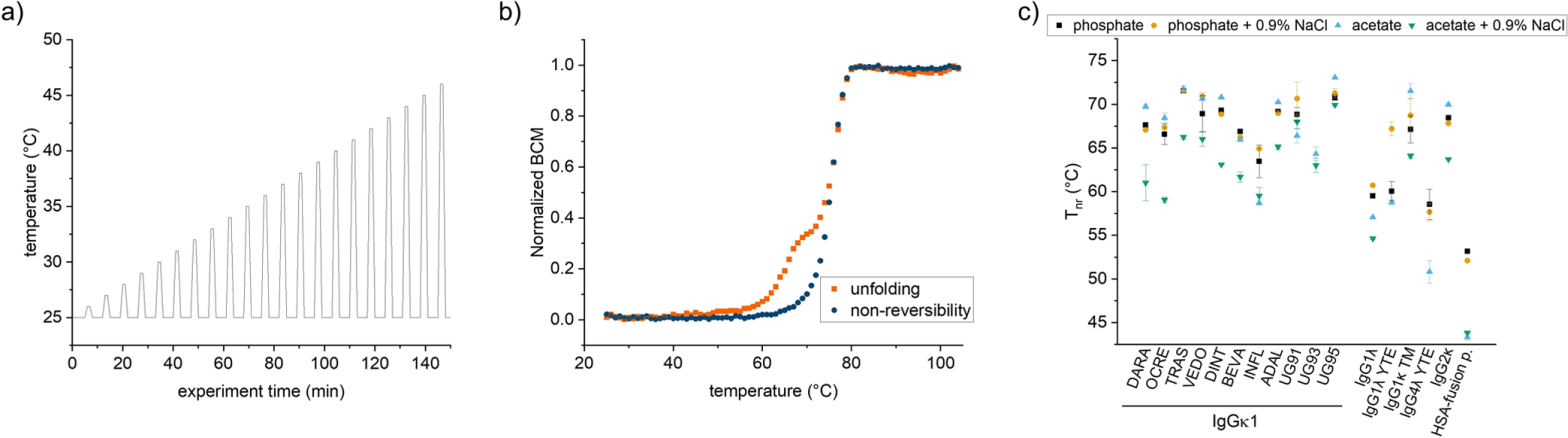
Modulated scanning fluorimetry in microwell plates. (a) Schematic incremental heating and cooling cycles. (b) Exemplary unfolding and nonreversibility curve of a model mAb UG95 in acetate with 0.9% NaCl. (c) T_nr_ of 17 model proteins in four conditions. Mean of triplicates with SD.

We tested whether the intrinsic DSF in microwell plates can be adapted to MSF experiments. To achieve this, we used a custom AutoIT script to perform incremental heating and cooling cycles (Figure 9a), and a custom Python script to process the measurement files (see the Methods section for more details). Using this approach, we were able to obtain the unfolding and nonreversibility curves of a range of model proteins (Figures 9b and S5). We then determined the nonreversibility onset temperatures (T_nr_) (Figure 9c). The model antibody UG95 in acetate buffer has the highest T_nr_ (73 °C), while the HSA-fusion protein exhibits the lowest T_nr_ values (Figure 9b).

Overall, the intrinsic DSF can easily be adapted to MSF experiments, expanding the capability of the method to the measurement of 384 samples in parallel.

## Discussion

Fluorescence-based techniques such as DSF have become essential for the characterization of biotherapeutic candidates and formulations. Here, we investigated and expanded the capabilities of intrinsic DSF in microwell plates. The approach presents a significant advancement in terms of increased throughput (parallel measurement of 384 samples), reduced consumable costs, and straightforward automation. Using diverse model proteins, we proved that the approach is comparable to gold-standard methods such as μDSC and CD used for determination of protein unfolding. However, the big advantage of intrinsic DSF is the possibility to measure up to 384 samples in parallel with only 10 μL per sample. The wide range of protein concentrations that can be accommodated within the same intrinsic DSF experiment is another major advantage. The lowest protein concentration that can be used depends on the protein and the sample (number of tryptophan residues, complexity of the unfolding and photophysical properties), but we showed with two different model molecules (HEWL and a monoclonal antibody) that the melting transitions of the proteins can be detected using protein concentrations as low as 0.05–0.1 mg/mL. A further advantage of intrinsic DSF is its compatibility with samples containing different impurities and chemicals (e.g., pDNA, DMSO, EDTA, polysorbates). In contrast, extrinsic DSF with dyes such as SyproOrange cannot be performed reliably on protein samples containing frequently used concentrations of surfactants such as polysorbates.

We applied the intrinsic DSF to a wide range of antibodies and protein modalities. This testifies to the versatility of the technique that can be used for candidate and formulation screening. A further application of intrinsic DSF that we demonstrated with an ACE2-Fc fusion protein and an ACE2 inhibitor is the detection of stabilizing protein–ligand interactions.

The versatility and robustness of the intrinsic DSF are matched with an impressive throughput that can easily reach several thousand samples per day, especially when combined with automation techniques such as described in Hansel et al. (2023).^22^ Such throughput surpasses the current small-scale protein production capabilities in a wet lab. Therefore, advances in high-throughput protein production techniques will be required to match the efficiency of intrinsic DSF for stability data collection.

It is essential that intrinsic DSF is combined with straightforward data analysis software such as MoltenProt.^23^ A major obstacle for fast DSF data analysis is the complexity of the unfolding curves. For example, we observed that even IgG1k antibodies that differ only in the variable domains show very different unfolding behavior exhibiting one or multiple transitions (Figure S3). New analysis algorithms should be able to automatically process the DSF data to find the best fitting model (e.g., two-state, three-state) and optimize the fit for each curve to yield reliable numerical data such T_onset_ and *T*_m_s.

There have been significant improvements in both extrinsic and intrinsic DSF recently. For example, the use of different reporter dyes has opened new directions in DSF applications for the analysis of protein stability, dynamics, and interactions.^24^ It is expected that the intrinsic DSF also develops further to provide additional information on protein dynamics and interactions. For example, analysis of the red edge excitation shift (REES) phenomena^25,26^ and fluorescence polarization measurements^27^ could provide another layer of information in next-generation DSF devices.

Microwell-plate-based intrinsic DSF can also be used for long isothermal experiments at elevated temperatures (Figure S6). Although, the isothermal kinetic analysis of protein unfolding is out of the scope of this article, fitting such data obtained from hundreds of samples in parallel can yield valuable kinetic data.^13,14^

The technical capabilities of intrinsic DSF have already developed immensely in the past ten years. As we demonstrate, it is now also possible to use very different heating ramps and complex heating programs on hundreds of samples in parallel. As a result, new applications such as MSF could be developed. MSF yields information on the temperature that starts to cause irreversible structural changes in a protein (T_nr_) which usually is the onset of protein aggregation. For example, T_nr_ correlates with *T*_agg_ determined with DLS.^17^ Techniques such as MSF are performed on the same equipment used for intrinsic DSF but provide orthogonal stability information. As a result of these and similar technological improvements, the intrinsic DSF instruments become more and more versatile and find more applications for protein characterization.

In summary, the presented microwell plate-based DSF/MSF methodologies merge into a versatile platform for comprehensive protein stability analysis with a single device.

## Supporting Information Available

Figure S1 - Fluorescence spectra comparing measurements in microwell plates and quartz cuvette; Figure S2 - Fluorescence background created in microwell plates by buffer containing EDTA, DMSO, or pDNA; Figure S3 - Unfolding curves of different IgGκ1 antibodies in intrinsic DSF; Figure S4 - Differences in T_onset_ for HEWL depending on concentration and ramp rate; Figure S5 -Unfolding and nonreversibility curves from MSF measurements; Figure S6 - Fluorescence changes during isothermal incubation of proteins; Table S1 - Number oftryptophan residues in the used proteins; Table S2 - Goodness of the fit for intrinsic DSF measurements (PDF)
