# Supplementary material for: Intrinsic Differential Scanning Fluorimetry for Protein Stability Assessment in Microwell Plates
Source: Mol Pharm. 2025 Feb 7;22(3):1697–706. doi: 10.1021/acs.molpharmaceut.4c01496 (PMC11881137; doi:10.1021/acs.molpharmaceut.4c01496)
Supplement: Supplementary file 1 — mp4c01496_si_001.pdf [file mp4c01496_si_001.pdf]

Supporting Information

Intrinsic differential scanning fluorimetry for protein stability assessment in microwell plates

Michaela Cohrs[1], Alastair Davy[2], Manon Van Ackere[1], Stefaan De Smedt[1], Kevin Braeckmans[1], Markus Epe[2], Hristo L. Svilenov[1,3 *]

[1] Laboratory of General Biochemistry and Physical Pharmacy, Ghent University, Ottergemsesteenweg 460, 9000 Ghent, Belgium

[2] Protein Stable Ltd., 21 Mole Business Park, KT22 7BA Leatherhead, UK, markus.epe@photophysics.com

[3] Biopharmaceutical Technology, TUM School of Life Sciences, Technical University of Munich, Emil-Erlenmeyer-Forum 5, 85354 Freising, Germany hristo.svilenov@tum.de

* To whom correspondence should be addressed:

hristo.svilenov@tum.de, https://orcid.org/0000-0001-5863-9569

phone: 0049 8161 71 2266

Figure S1

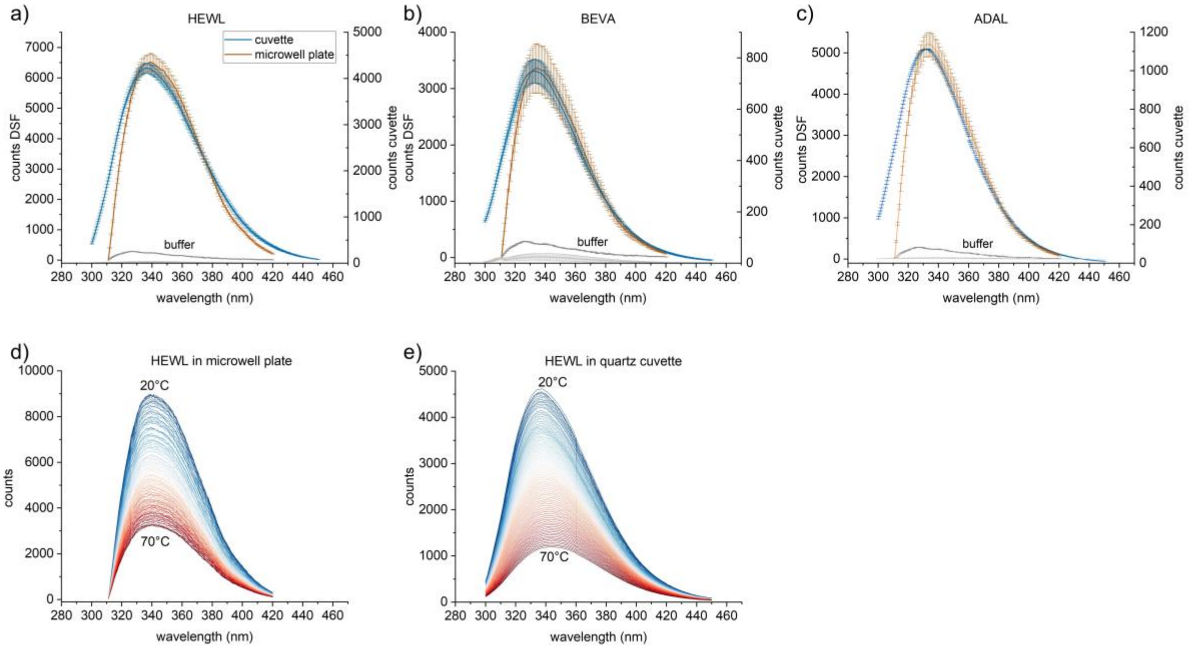

Figure S1. Emission spectra of **(a)** HEWL, **(b)** BEVA and **(c)** ADAL as 1 mg/ml in acetate buffer 20 mM, pH 5 in microwell plates vs quartz cuvette at 20 °C. Buffer as background is presented. Mean of triplicates with SD. **(d)** Spectral change during heating of 1 mg/ml HEWL in 20 mM acetate buffer pH 5 in a microwell plate and **(e)** in a quartz cuvette.

Figure S2

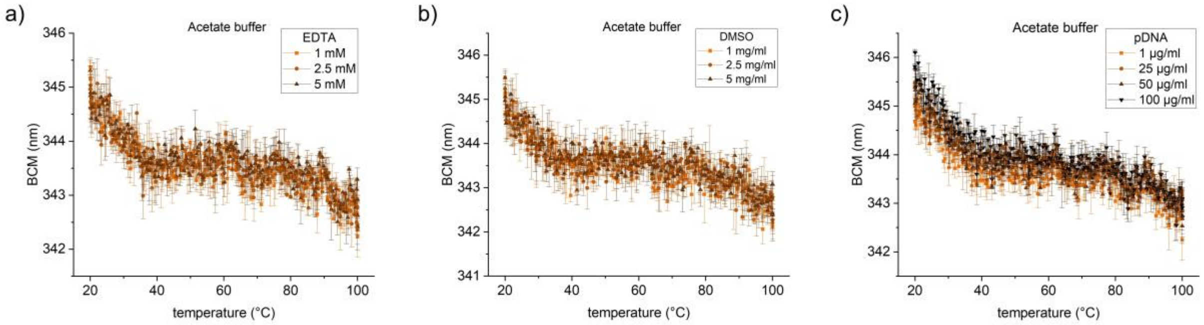

Figure S2. Background of acetate buffer 20 mM pH 5 under addition of **(a)** EDTA, **(b)** DMSO, and **(c)** pDNA. Mean of triplicates with SD.

Figure S3

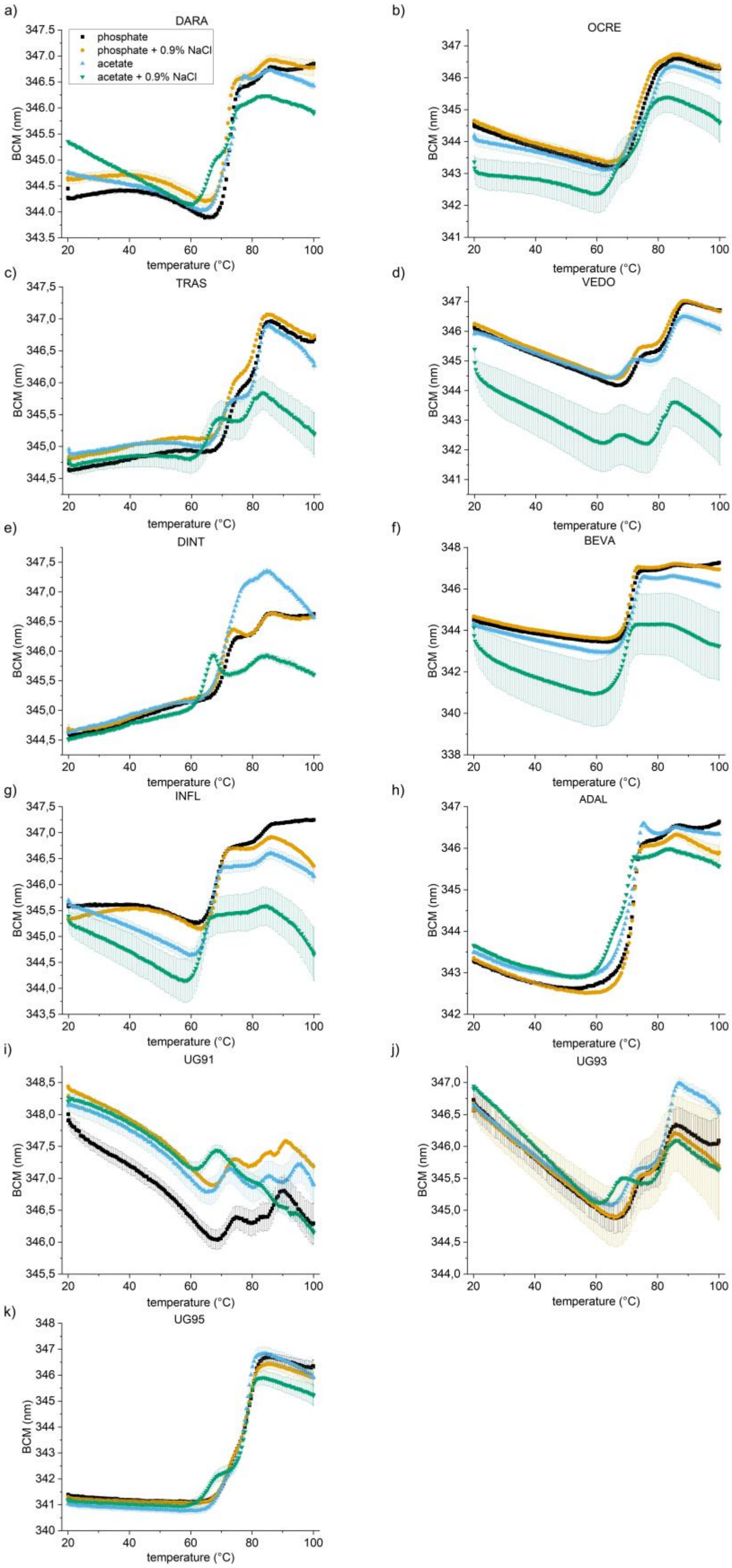

Figure S3. Unfolding of IgGκ1 during heating with 1 K/min. All samples are formulated at 0.5 mg/ml. Mean of triplicates with SD.

Figure S4

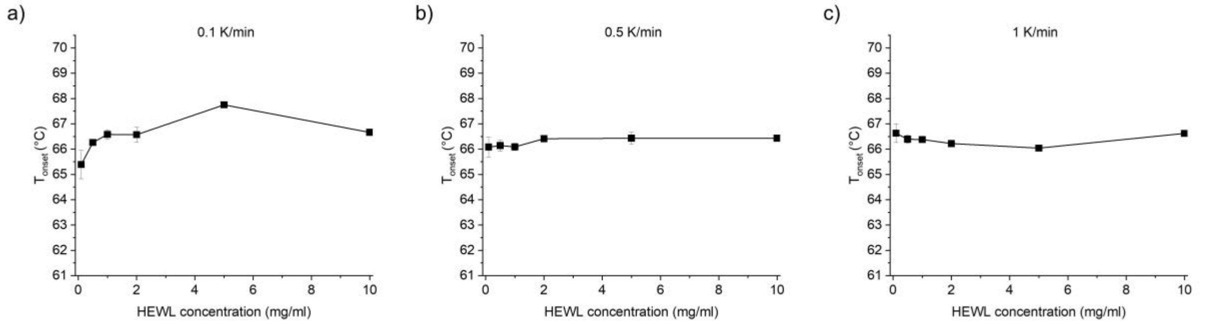

Figure S4. Differences in T_{onset} of HEWL caused by variations in ramp rate and protein concentration. 0.1-10 mg/ml HEWL in 10 mM phosphate buffer pH 7 measured with a ramp rate of **(a)** 0.1 K/min, **(b)** 0.5 K/min, and **(c)** 1 K/min. All values are mean of triplicates with SD.

Figure S5

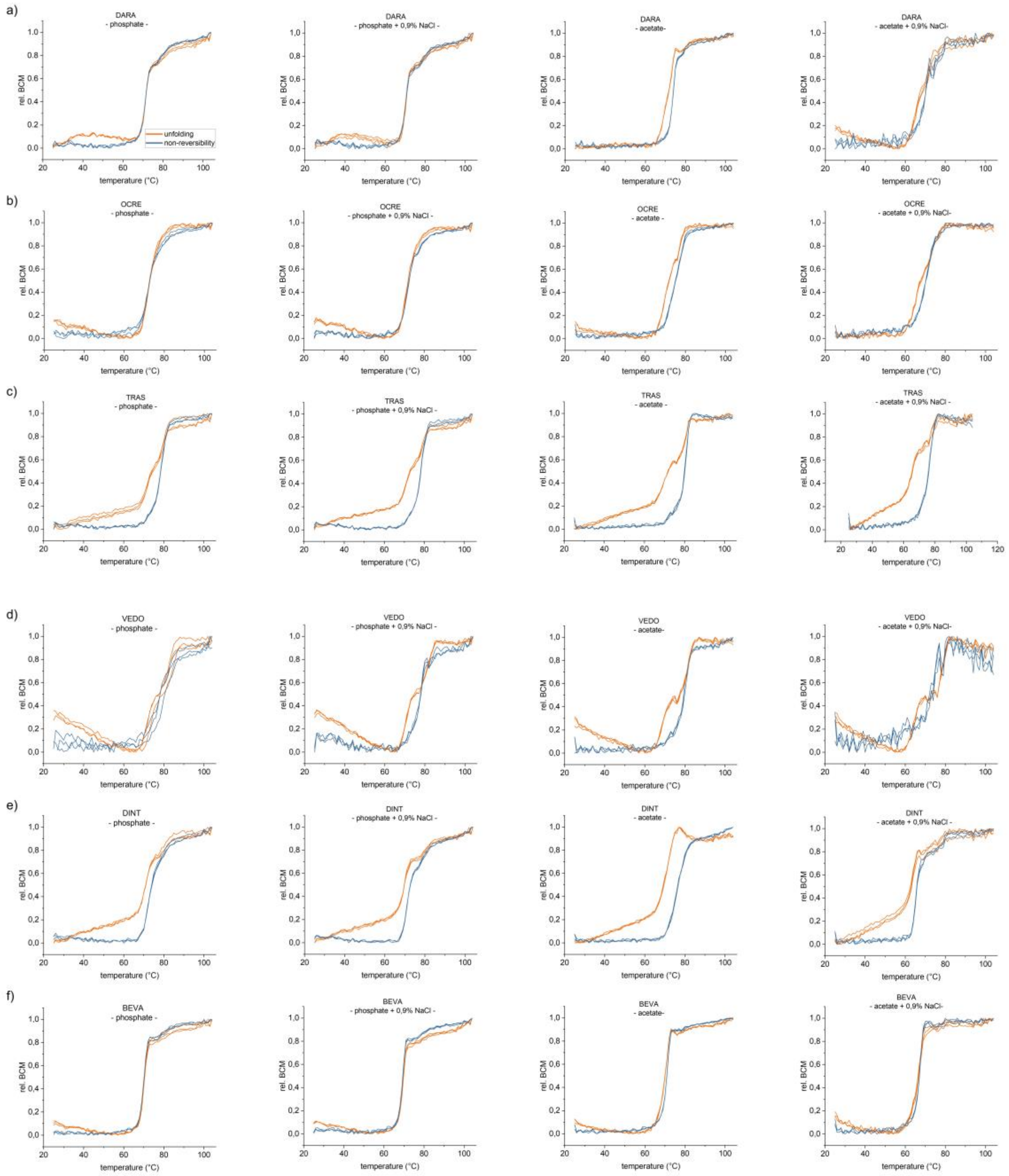

figure continues on next page

continued from previous page

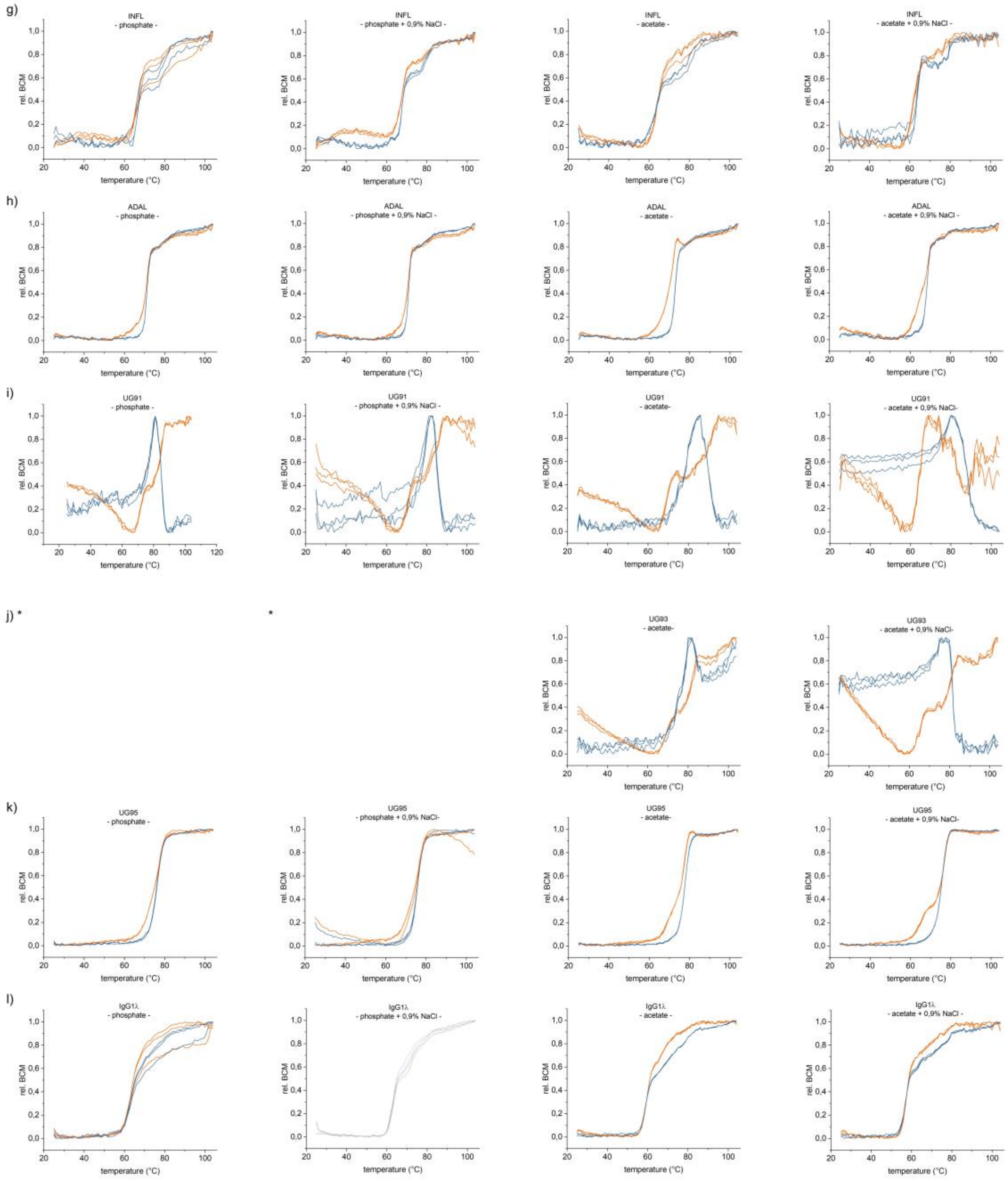

figure continues on next page

continued from previous page

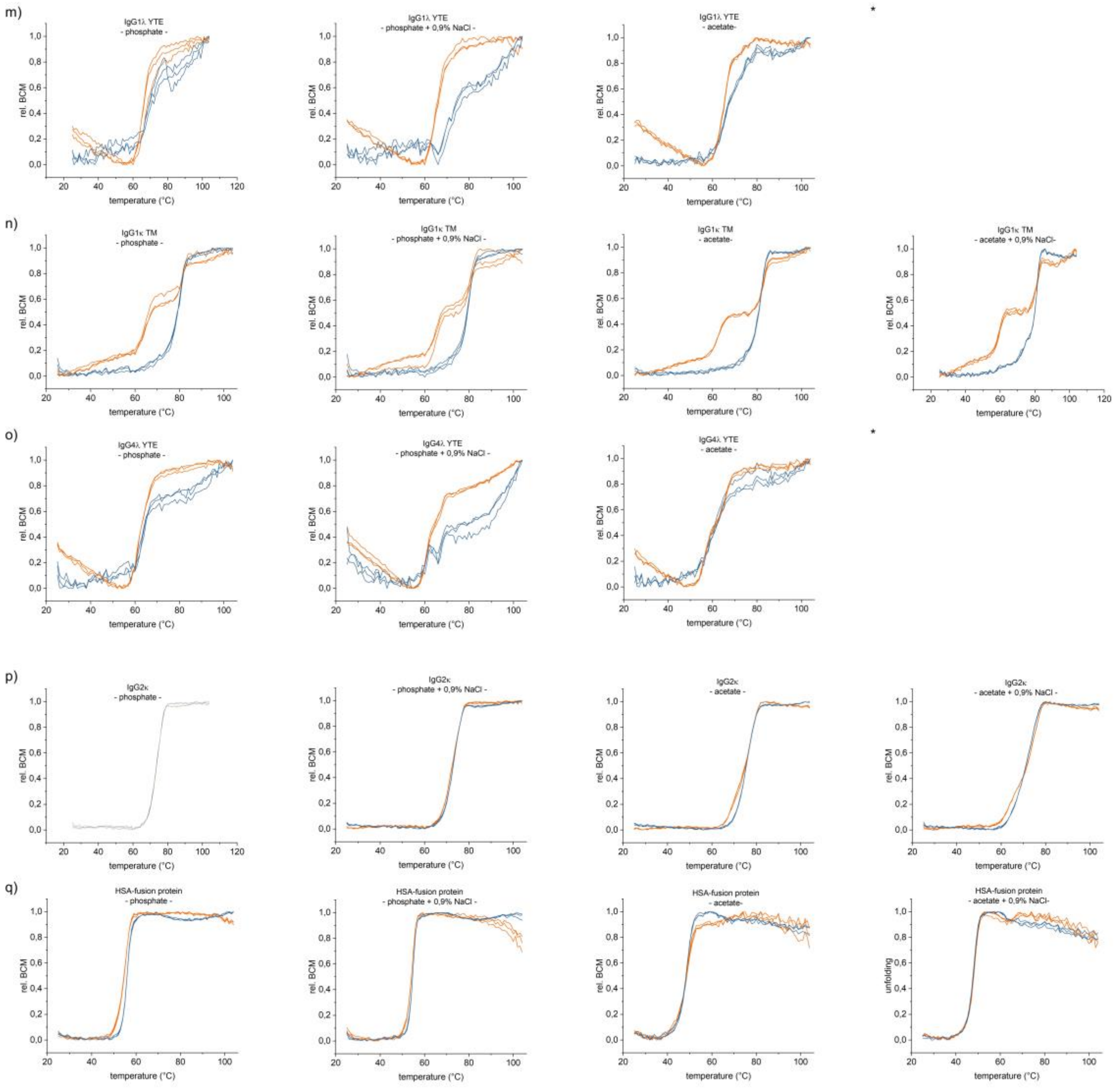

Figure S5. Unfolding (orange) and non-reversibility (blue) curves of triplicates shown. All samples were formulated at 0.5 mg/ml.

Figure S6

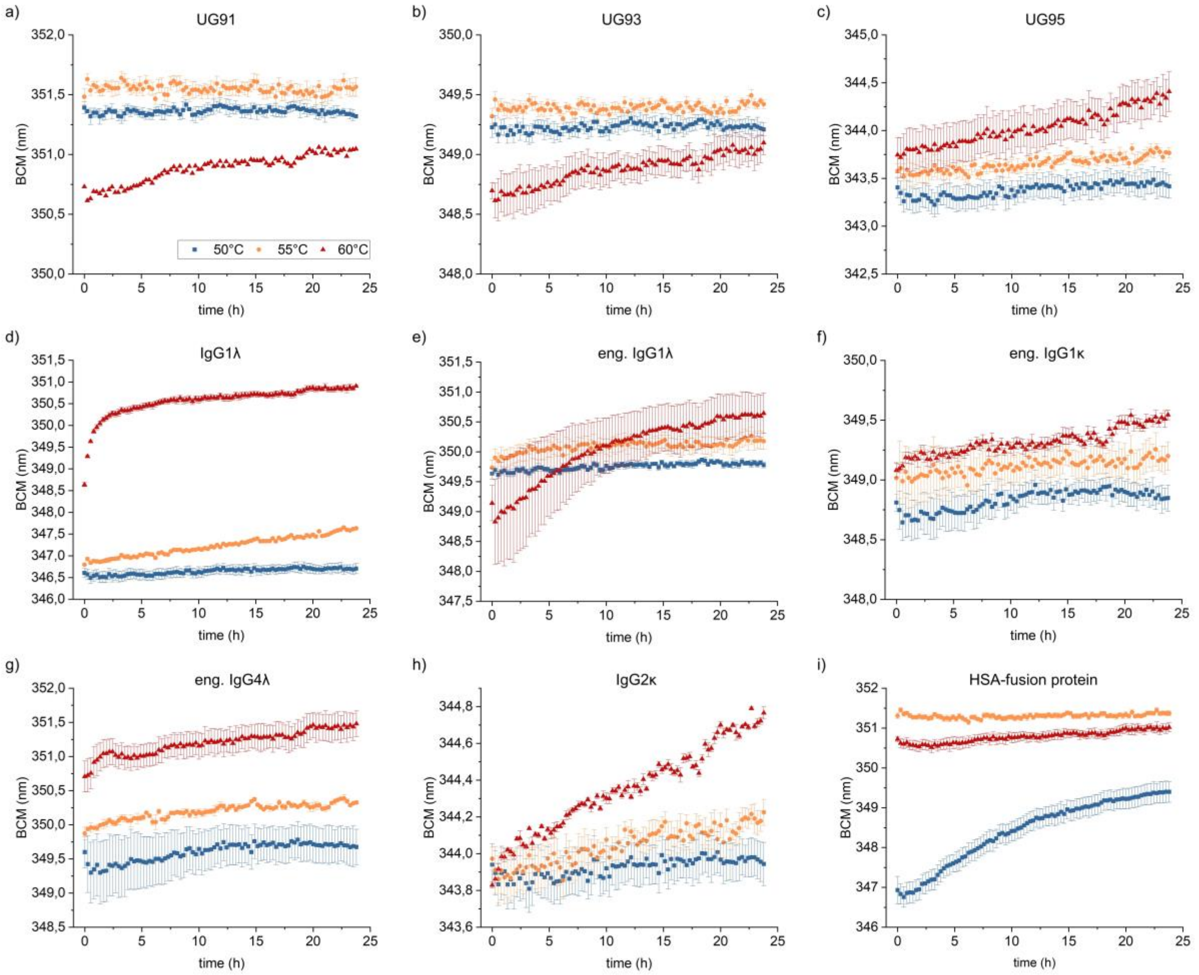

Figure S6. Unfolding during isothermal incubation at elevated temperatures followed by intrinsic fluorescence. All samples were diluted with 1x PBS, pH 7.4 to 1 mg/ml. Mean of triplicates with SD.

Table S1

**Protein**	**Sequence source**	**Trp count**
HEWL	UniProt, P00698	6
TRAS	Drugbank Online (Knox et al., 2024)	22
BEVA	Drugbank Online (Knox et al., 2024)	26
ADAL	Drugbank Online (Knox et al., 2024)	22
DARA	Drugbank Online (Knox et al., 2024)	24
OCRE	Drugbank Online (Knox et al., 2024)	24
VEDO	Drugbank Online (Knox et al., 2024)	24
DINT	KEGG DRUG, D10559	20
INFL	PDBe, 5vh5|A, 5vh4|B L, 5vh4|A H	24
rHGH	Drugbank Online (Knox et al., 2024)	1

Table S1. Tryptophan count per protein with source of sequence.

Table S2

Figure	Fit	Chi-Square
1f	White plate	0.5864
1f	Black plate	0.6558
Tabel 1	HEWL in phosphate buffer	1.2235
	BEVA in phosphate buffer	1.8592
	ADAL in phosphate buffer	1.5875
	HEWL in acetate buffer	1.3184
	BEVA in acetate buffer	1.5427
	ADAL in acetate buffer	1.2765
5 b,c	DARA phosphate	0.0176
	DARA phosphate + 0.9% NaCl	0.0131
	DARA acetate	0.0065
	DARA acetate + 0.9% NaCl	0.0033
	OCRE phosphate	0.0032
	OCRE phosphate + 0.9% NaCl	0.0039
	OCRE acetate	0.0050
	OCRE acetate + 0.9% NaCl	0.0141
	TRAS phosphate	0.0208
	TRAS phosphate + 0.9% NaCl	0.0212
	TRAS acetate	0.0265
	TRAS acetate + 0.9% NaCl	0.0283
	VEDO phosphate	0.0026
	VEDO phosphate + 0.9% NaCl	0.0027
	VEDO acetate	0.0021
	VEDO acetate + 0.9% NaCl	0.0100
	DINT phosphate	0.0095
	DINT phosphate + 0.9% NaCl	0.0174
	DINT acetate	0.0297
	DINT acetate + 0.9% NaCl	0.0651
	BEVA phosphate	0.0096
	BEVA phosphate + 0.9% NaCl	0.0064
	BEVA acetate	0.0091
	BEVA acetate + 0.9% NaCl	0.0177
	INFL phosphate	0.0104
	INFL phosphate + 0.9% NaCl	0.0231
	INFL acetate	0.0071
	INFL acetate + 0.9% NaCl	0.0120
	ADAL phosphate	0.0157
	ADAL phosphate + 0.9% NaCl	0.0192
	ADAL acetate	0.0424
	ADAL acetate + 0.9% NaCl	0.0048
	UG91 phosphate	0.0171
	UG91 phosphate + 0.9% NaCl	0.0073
	UG91 acetate	0.0093
	UG91 acetate + 0.9% NaCl	0.0109
	UG93 phosphate	0.0046
	UG93 phosphate + 0.9% NaCl	0.0046
	UG93 acetate	0.0115
	UG93 acetate + 0.9% NaCl	0.0071
	UG95 phosphate	0.0166
	UG95 phosphate + 0.9% NaCl	0.0200
	UG95 acetate	0.0389
	UG95 acetate + 0.9% NaCl	0.0379
7b	100:1	1.8627
	20:1	1.2255
	10:1	1.4962
	2:1	1.6874

	1:1	1.7551
	1:2	1.8392
	1:10	2.0309
	1:20	1.9540
8a	HEWL 0.1 mg/ml	0.0025
	HEWL 0.5 mg/ml	0.0015
	HEWL 1 mg/ml	0.0012
	HEWL 2 mg/ml	0.0010
	HEWL 5 mg/ml	0.0080
	HEWL 10 mg/ml	0.0086
8b	HEWL 0.1 mg/ml	0.0038
	HEWL 0.5 mg/ml	0.0010
	HEWL 1 mg/ml	0.0007
	HEWL 2 mg/ml	0.0010
	HEWL 5 mg/ml	0.0041
	HEWL 10 mg/ml	0.0004
8c	HEWL 0.1 mg/ml	0.0018
	HEWL 0.5 mg/ml	0.0010
	HEWL 1 mg/ml	0.0005
	HEWL 2 mg/ml	0.0006
	HEWL 5 mg/ml	0.0010
	HEWL 10 mg/ml	0.0003
8d	HEWL 0.5 mg/ml	0.0028
	HEWL 1 mg/ml	0.0016
	HEWL 2 mg/ml	0.0011
	HEWL 5 mg/ml	0.0007
8e	HEWL 0.5 mg/ml	0.0127
	HEWL 1 mg/ml	0.0039
	HEWL 2 mg/ml	0.0015
	HEWL 5 mg/ml	0.0006
8f	HEWL 0.5 mg/ml	0.0032
	HEWL 1 mg/ml	0.0018
	HEWL 2 mg/ml	0.0010
	HEWL 5 mg/ml	0.0005

Table S2. Goodness of the fit presented as Chi-Square sorted for each Figure.

Reference

Knox, C., Wilson, M., Klinger, C. M., Franklin, M., Oler, E., Wilson, A., Pon, A., Cox, J., Chin, N. E. (Lucy), Strawbridge, S. A., Garcia-Patino, M., Kruger, R., Sivakumaran, A., Sanford, S., Doshi, R., Khetarpal, N., Fatokun, O., Doucet, D., Zubkowski, A., … Wishart, D. S. (2024). DrugBank 6.0: the DrugBank Knowledgebase for 2024. *Nucleic Acids Research*, *52*(D1), D1265–D1275. https://doi.org/10.1093/nar/gkad976
